# Interactive Attention Learning on Detection of Lane and Lane Marking on the Road by Monocular Camera Image

**DOI:** 10.3390/s23146545

**Published:** 2023-07-20

**Authors:** Wei Tian, Xianwang Yu, Haohao Hu

**Affiliations:** 1 Tongji University, Shanghai 201804, China; yuxianwang@tongji.edu.cn; 2 Institute of Measurement and Control Systems, Karlsruhe Institute of Technology, 76131 Karlsruhe, Germany; haohao.hu@kit.edu

**Keywords:** interactive attention learning, lane segmentation, lane marking detection

## Abstract

Vision-based identification of lane area and lane marking on the road is an indispensable function for intelligent driving vehicles, especially for localization, mapping and planning tasks. However, due to the increasing complexity of traffic scenes, such as occlusion and discontinuity, detecting lanes and lane markings from an image captured by a monocular camera becomes persistently challenging. The lanes and lane markings have a strong position correlation and are constrained by a spatial geometry prior to the driving scene. Most existing studies only explore a single task, i.e., either lane marking or lane detection, and do not consider the inherent connection or exploit the modeling of this kind of relationship between both elements to improve the detection performance of both tasks. In this paper, we establish a novel multi-task encoder–decoder framework for the simultaneous detection of lanes and lane markings. This approach deploys a dual-branch architecture to extract image information from different scales. By revealing the spatial constraints between lanes and lane markings, we propose an interactive attention learning for their feature information, which involves a Deformable Feature Fusion module for feature encoding, a Cross-Context module as information decoder, a Cross-IoU loss and a Focal-style loss weighting for robust training. Without bells and whistles, our method achieves state-of-the-art results on tasks of lane marking detection (with 32.53% on IoU, 81.61% on accuracy) and lane segmentation (with 91.72% on mIoU) of the BDD100K dataset, which showcases an improvement of 6.33% on IoU, 11.11% on accuracy in lane marking detection and 0.22% on mIoU in lane detection compared to the previous methods.

## 1. Introduction

Lanes and lane markings are essential road information for intelligent driving vehicles. The lane marking detection aims to accurately locate road elements like lane lines, crosswalks, and stop zones, while the lane detection focuses on segmenting lane-level areas where vehicles can drive on the road. Due to the low cost and the high representability of scene information, optical sensors and instruments, such as the on-board camera, are widely adopted for road information perception. By applying lane and lane marking detection approaches, visual features of road symbols, arrows, lane markings, pedestrian crosswalks, and vehicle drivable areas, etc., are extracted from the image. These features are indispensable for both high-level autonomous driving or for general ADAS-assisted driving systems. They can be considered as elements in the high-definition map construction, or further converted into the information required by the planning and control system, to assist the driving behavior of vehicles, especially in applications such as adaptive cruise control (ACC), driving route navigation, lane keeping assistance (LKA), etc., thus ensuring driving safety and reliability [1,2,3].

Generally, the detection of lanes and lane markings can be classified in two categories: the traditional paradigm [4,5], and the deep learning paradigm [6,7,8]. Traditional methods rely on hand-crafted features and sophistically designed rules to manipulate the information from color space or shape structure to detect lanes and lane markings. Due to their poor feature representability, these methods are only limited scalable to varied scenes. In recent years, the deep learning approaches in computer vision have achieved remarkable progress, especially in object detection and semantic segmentation tasks. Since the lanes and lane lines are normally made with inherent long and thin shapes and even irregular ones, the difficulty lies in the exploration of effective representation learning of their complex structures.

In current studies, the detection of lanes and lane markings are considered as two individual tasks. The lane detection is typically interpreted as a pixel-wise semantic segmentation problem while the lane marking can be predicted with various formulations such as instance segmentation [9,10], point regression [11,12], curve parameter estimation [13,14,15], etc. Although both tasks have witnessed persistent progress in recent years, especially on public benchmarks [9,16,17], one fact that has been neglected is that the information of lanes and lane markings on the road scene are complementary to each other. For instance, on structured roads, the associated lane lines can be used to identify the lane boundaries while in scenarios where lane lines or crosswalks are partially missing or broken (due to occlusion), they can still be inferred by the width of the lane. Thus, the detection of lane and lane marking are inherently correlated due to their spatial connectivity. In real driving scenarios, the detection robustness of a single task is poor, and it can be easily disturbed by the disappearance of visual markings, e.g., due to occlusions. However, leveraging the spatial connectivity between the lane and lane marking, the detection robustness can be improved by modeling this internal connection, which has not been studied in existing methods.

**Figure 1:**
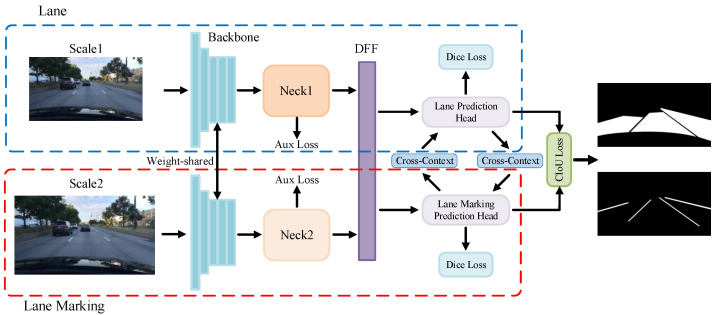
Overview of the proposed interactive attention learning model on detection of lanes and lane markings. Our model is decomposed into two branches, each consisting of a backbone, a neck and a prediction head with information interactively learned by the DFF, Cross-Context and CIoU loss module.

Inspired by this fact, this work proposes a novel multi-task encoder–decoder architecture for simultaneous detection of lane and lane marking based on the spatial relationship between them. Specifically, this architecture interprets both tasks as semantic segmentation problems and adopts multi-scale inputs. In the encoding stage, it employs a feature interactive attention structure, namely the Deformable Feature Fusion (DFF) module, to calculate a relative offset between feature encodings of lane and lane marking, supporting a deformable convolution operation for interaction. The decoding for lanes and lane markings is conducted separately, and a Cross-Context module is used to transfer the decoding information between them. To further exploit their spatial relationship, we add a Cross Intersection-over-Union (CIoU) loss at the output of the lane and lane marking decoders. Thus, the interactive learning has been leveraged at the encoding stage, the decoding stage, and the outputs. Furthermore, we deploy a Focal-style loss weighting to adaptively set loss weights at different pixel locations to alleviate the data imbalance problem in lane and lane marking segmentation. The whole framework is shown in Figure 1. By testing on the BDD100K dataset, our method manifests a state-of-the-art performance on the lane detection (with 32.53% lane IoU, 81.61% lane accuracy) and the drivable area segmentation (with 91.72% drivable mIoU) tasks.

The main contributions of the proposed work are summarized as follows:

• We propose a novel multi-task encoder–decoder architecture. It is the first to introduce the concept of interactive attention learning into the joint detection of lane and lane marking.
• We propose the DFF module in calculating discriminative encoding features, and employ the Cross-Context module to transfer information between prediction heads, thus shifting the focus of learning on the spatial correlation between lane and lane marking.
• We propose an enhanced loss function with a CIoU loss to emphasize the lane and lane marking interaction and an adaptive pixel-level loss weighting to alleviate data imbalance.

## 2. Related Works

In this section, we give a brief review about related works in terms of the task setup and architecture design of our work, i.e., the lane marking detection approaches, the lane detection approaches, and multi-task approaches.

### 2.1. Lane Marking Detection

As aforementioned, traditional lane marking detection approaches generally rely on sophisticated model design and hand-crafted features, involving color conversion [4], combination of Kalman and particle filter [18], bar filter [19] and Hough transform [5]. These approaches directly output lane segments, which are further post-processed to remove false positives and grouped to form the lane markings. Aly [20] proposed a robust real-time lane marking detection method, which first generated a top view image by projection transform and then extracted lane markings using a bar filter and a simple Hough transform. Assidiq et al. [21] detected edges with the Canny operator and extracted line features through the Hough transform. The lane marking was obtained by line fitting to selected pixels. However, limited by the poor feature representation, traditional methods show inrobustness in complex scenarios, such as with broken lane markings or occlusion by vehicles and pedestrians.

In recent years, the deep learning technique has significantly boosted the lane marking detection performance. According to the modeling strategy, such approaches can be classified into four categories: segmentation-based, anchor-based, row-wise detection, and parametric prediction methods. The segmentation-based methods commonly adopt the semantic segmentation or instance segmentation to make pixel-wise predictions [6,9,10]. Supervised by a sufficient amount of labeled data, these approaches show advantages in detecting various kinds of lane markings. The aerial LaneNet [22] proposed a fully convolutional neural network in a symmetrical structure, which is enhanced by wavelet transform for lane marking segmentation in aerial imagery. Guan et al. [23] incorporated the attention mechanism into FPN networks to extract better road marking segmentation results from high resolution UAV images. The anchor-based methods leverage the anchor concept from traditional object detection, but differ from them by taking into account the shape characteristics of lane markings. For instance, the PointLaneNet [7] and CurveLane-NAS [24] define anchors with vertical lines, while the Line-CNN [11] and LaneATT [12] adopt the Line Proposal Unit, which resembles the Region Proposal network (RPN) of the Faster-RCNN [25]. The row-wise detection approaches make full use of the prior shape of lane markings as well as their spatial distribution characteristics. They divide the image into grids and make row-wise predictions to locate the lane markings [26,27,28]. In contrast, the parametric prediction methods define lane markings (especially lane lines) as curve functions with a set of parameters, such as polynomials [13,14], and Bézier curves [15]. Their interpretations are significantly different from the above-mentioned methods and the corresponding curve parameters are difficult to learn. In addition, to solve the problem of difficult scenes for lane marking detection such as occlusion and low-visibility, Wang et al. [29] proposed a dynamic data augmentation framework based on imitating real scenes.

### 2.2. Lane Detection

The task of lane detection is also known as the drivable area detection, which is mainly classified as a segmentation task at present. As a result of the great successes of the deep learning, many methods based on semantic segmentation and instance segmentation can be transferred to the drivable area detection. The FCN [30] is the first work to introduce the fully convolutional network to semantic segmentation, which makes CNN-based methods widely applicable for lane detection. The UNet [31] further constructs an encoder–decoder framework to extract lane semantic information from high-dimensional features. The DeepLabV3 [32] combines the atrous convolutions [33] with different artous rates to fuse the feature pyramid, namely ASPP, obtaining different receptive fields on feature maps. The PSPNet [34] proposes the pyramid pooling module for feature extraction of various scales, which enhances the accuracy of the model. It is also worth noting that both DeepLabV3 and PSPNet leverage the fusion of multi-scale feature information to improve the segmentation performance. He et al. [35] embedded the Swin transformer into the classical network (UNet) to improve the semantic segmentation performance for remote sensing images. Xie et al. [36] presented a segmentation method for RGB-D data and adopted the motion detection to improve the inference accuracy. Meyer et al. [37] expanded the Cityscapes dataset [38] by lane-level annotations and presented a novel lane detection pipeline, which used the stereo system to convert the front-view segmentation results into a form of 3D point cloud and projected it to the top-view. Sun et al. [39] proposed to leverage crowd-sourced GPS data to extract roads from an aerial image, which achieved improved road segmentation compared to previous works. Fontanelli et al. [40] performed lane detection in the front-view image and projected it to the top-view for the construction of the path, which is used to plan the future motion of the robot.

### 2.3. Multi-Task Approaches

Although previous studies have achieved excellent performance in a single detection or segmentation task, the multi-task architecture to process perception information is more friendly to practical applications. The goal of multi-task approaches is to establish a trade-off between the detection performance and the computational complexity by utilizing the shared feature information and model structure.

The MultiNet [41] first introduces a multi-task architecture into the autonomous driving perception task. The architecture adopts a shared backbone and three decoders to perform tasks of road segmentation, vehicle detection, and scene classification simultaneously. The DLT-Net [42] inherits the encoder–decoder architecture with a shared backbone and multi-task decoders. It transmits the information from the drivable area decoder, namely the context tensor, to both the lane marking decoder and the traffic object decoder, thus sharing the decoder information to a certain extent. The RBNet [43] proposes a multi-task neural network model for unified detection of road and road boundary, which combined the input image, road and road boundary as three nodes into a Bayesian network. Zhang et al. [44] considered the geometric constraint between the road and its boundaries and constructed interlinked sub-networks for overall performance improvement of both detection tasks. The RoadNet [45] develops a multi-task convolutional neural network to simultaneously make predictions of road boundaries, surfaces, and centerline based on the high-resolution images from remote sensing. The HYDRO-3D [46] incorporates object detection features with historical object tracking information to improve the performance of both tasks, which achieves robust object detection. Xia et al. [47] proposes a platform for automated driving system data acquisition and analysis, which presents a holistic pipeline for data processing based on connected automated vehicles. However, the exploration on the interaction between lane and lane marking information is still insufficient in the above-mentioned studies.

## 3. Methodology

Here, we present the proposed lane and lane marking detection architecture in detail, including a configured encoder, a DFF module, a decoder with a Cross-Context module, and loss functions with adaptive weights. Our code is publicly available at https://github.com/HerrYu123/IALaneNet, accessed on 1 December 2022.

### 3.1. Architecture Overview

The overall architecture as presented in Figure 1 is divided into two branches for lane and lane marking detection, respectively. Both branches have a similar structure, consisting of a scaled input image, a backbone, a contextual neck, and a decoder. The two branches are connected by three interaction modules, i.e., the DFF module for the encoder, the Cross-Context module for the decoder, and the Cross-IoU loss for the lane and lane marking outputs.

We employ a modified variant of the ConvNeXt [48] as our backbone, which generates lane and lane marking feature maps with an output stride of 8. The feature maps are processed by the neck network, i.e., the REcurrent Feature-Shift Aggregator (RESA) [10], which adopts 1 × 9 convolution kernels for the spatial feature aggregation in which the sliced feature map is shifted in horizontal and vertical directions. The aggregated feature maps from both branches are fused in the DFF module. The lane and lane marking segmentation are predicted by their output heads. In an effort to refine the results, we leverage a Cross-Context module, which consists of deformable convolutions to transfer complementary information between two prediction heads. The Cross-IoU loss is further used to enhance the interaction of segmentation results. Our entire network is end-to-end differentiable and both tasks can be jointly learned.

### 3.2. Encoder

#### 3.2.1. Input

As aforementioned, the multi-scale feature information plays an crucial role for the segmentation task. Thus, the encoder of our network takes two scales of an image as inputs, i.e., the 0.5× scale and 1× scale. Each branch is made up of a backbone network and a neck network, where only the backbone network is weight-shared. This is different from other popular multi-task methods [41,42,49], which only use one scale image and one shared backbone. Considering that lane markings are relatively smaller than lane areas and thus require representation with higher resolution, we empirically set the branch with a 0.5× scale of input image for lane inference and the other for lane marking inference, which enables the branches to extract features in appropriate scales for both tasks.

#### 3.2.2. Backbone

**Figure 2:**
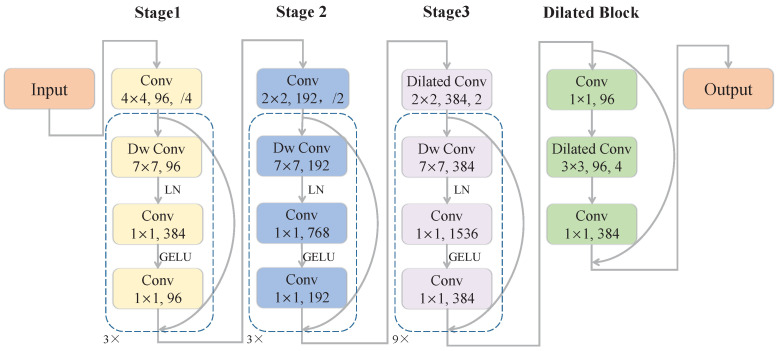
The structure of the backbone, which is configured based on the first three stages of the ConvNeXt [48] and one additional dilated block. Only the ConvNeXt-tiny is illustrated here.

The ConvNeXt [48] is used as the backbone of our architecture due to its outstanding performance in object detection tasks. However, the 1/32 downsampling layer in the naive ConvNeXt discards too much spatial information. As depicted in Figure 2, we simply replace the ordinary convolution in the downsampling layer of the third stage with atrous convolution and substitute the layers in the fourth stage for a dilated block to configure the backbone with an output stride of 8, which enables the following modules to extract scene features from a higher spatial resolution.

#### 3.2.3. Neck

The neck is used to extract contextual information from the feature maps, which are generated by the backbone. Here, we simply adopt the REcurrent Feature-Shift Aggregator modules for the neck network, as they are more efficient in gathering spatial information horizontally and vertically compared to other mainstream methods.

### 3.3. Deformable Feature Fusion Module

**Figure 3:**
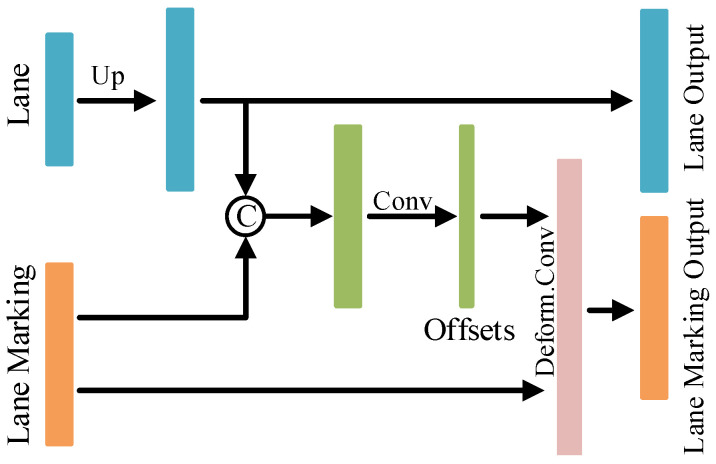
Architecture of the Deformable Feature Fusion Module.

Since the lane markings are typically in very thin and complex shapes, the learning of their accurate localization becomes more challenging. To address the above problem, here we propose the Deformable Feature Fusion Module, dubbed as DFF, illustrated in Figure 3. Such a module fuses features from the lane branch to assist the spatial information learning of lane markings, which is motivated by their strong spatial correlations. Specifically, the 0.5× scale lane feature map is fed into a 1 × 1 convolution operation and then recovered to the normal scale by a 2× bilinear interpolation upsampling. It is further concatenated with the 1× scale lane marking feature map. The interaction between the lane and lane marking is interpreted in a deformable convolution form, where the concatenated feature map will be leveraged to learn the offsets of the convolution. Thus, the spatial information from both encoders can be interactively learned by this module.

To ensure the spatial information correctly embedded into the input feature map of the DFF module, we simply add an auxiliary semantic segmentation branch to the output of each neck during the training process. These auxiliary segmentation branches are supervised by groundtruth labels of the lane and lane marking. This supervision can also be considered as a generalized form of residual learning. By setting up such supervision for shallow layers, the model could learn the basic semantic features in advance while the subsequent heads could focus on the learning of high-level information.

### 3.4. Decoder

As mentioned above, we cast the lane and lane marking detection as semantic segmentation tasks performed in two separate heads. However, we empirically found that the lane and lane marking have distinct characteristics. First, compared to the lane area, the slender lane marking is much more complicated to segment and thus it requires a higher processing complexity. Second, we noticed that the lane features are more suitable to decode from the shallower layers of the model than the lane marking features. Hence, the setup of the decoder is as follows.

#### 3.4.1. Lane Marking Prediction Head

Taking into account the slender characteristic of the lane markings, we adopt the Bilateral Up-Sampling Decoder (BUSD) [10] for decoding the lane marking features. The BUSD is able to combine the coarse grained feature and fine detailed feature in upsampling stage, which are extracted by direct bilinear interpolation and transpose convolution, respectively.

#### 3.4.2. Lane Prediction Head

Since the semantic area of the lane is larger and easier to detect compared to that of lane marking, we use a 1×1 output convolution and three standard semantic upsample modules, each consisting of a 3×3 convolution, a batch norm, and a ReLU function.

#### 3.4.3. Cross-Context Module

This Cross-Context mainly focuses on the pixel information located adjacent to the lane and lane marking, which is motivated by the their tight spatial connectivity. Since no attention is needed for the global features, we choose a convolution block to directly aggregate the information of local pixels. Taking into account the shape characteristics of lane and lane marking, the deformable CNNs [50] are leveraged in the Cross-Context module to implicitly establish a feature mapping for transferring the complementary information between two prediction heads, so that the decoding outputs can be reciprocally refined.

**Figure 4:**
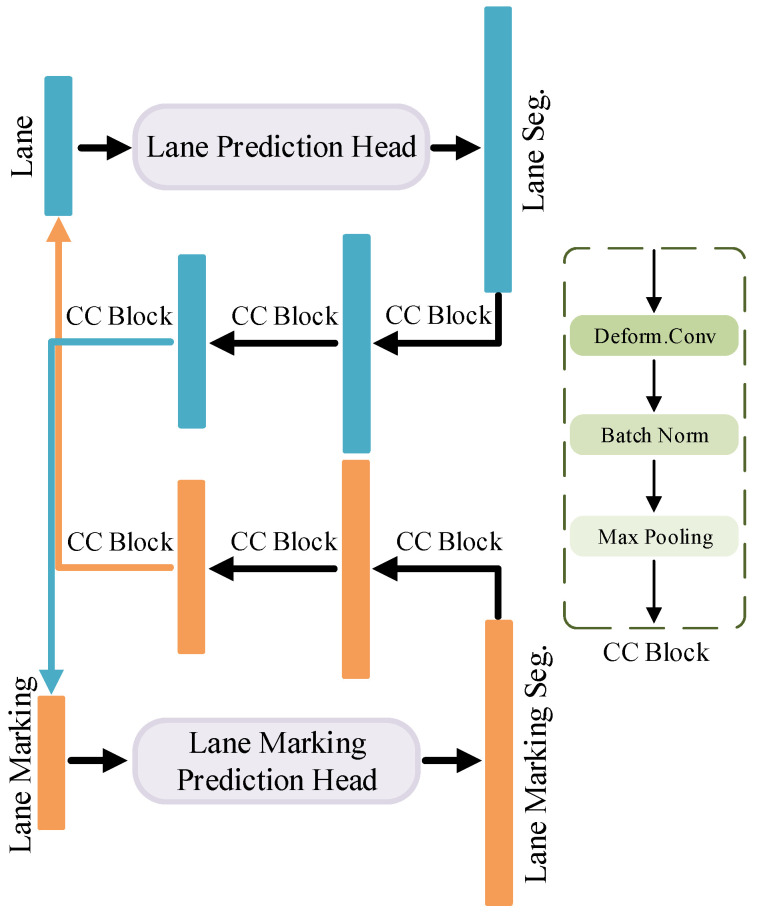
Architecture of the Cross-Context Module for decoding.

As illustrated in Figure 4, feature maps are first fed into the lane and lane marking prediction heads. The predicted segmentation maps are processed by the Cross-Context module and added to the input feature maps of each branch, respectively. The summed feature maps then pass the lane and lane marking decoder for the second time to obtain the final segmentation results. Thus, the Cross-Context module is able to draw the supplementary information from one decoder to another. Specifically, one Cross-Context module consists of three Cross-Context blocks for lane prediction head to lane marking prediction head, and another three for lane marking prediction head to lane prediction head. The Cross-Context block is made up of deformable convolution, batch normalization and max pooling. The Cross-Context module is expected to enhance the detection performance for both lane and lane marking with such a feature interaction of decoding information. Since the forward computation is performed twice in the decoders, it can be considered as a coarse-to-fine optimization.

### 3.5. Loss Function

#### 3.5.1. Segmentation Dice Loss

As a general consensus, the Dice loss [51] comes from the Dice coefficient and is proposed in the segmentation for alleviating the data imbalance problem by adjusting the training gradient distributions of positive and negative samples. Given semantic groundtruth labels [$y_{i}^{1},y_{i}^{2},\ldots ,y_{i}^{C}$] of *C* classes and the predicted probabilities [$p_{i}^{1},p_{i}^{2},\ldots ,p_{i}^{C}$] for the *i*-th pixel, the Dice loss can be formulated as

(1) $$L_{i}=1-\frac{1}{C}\sum_{k=1}^{C}\frac{2\sum_{i}^{N}y_{i}^{k}p_{i}^{k}}{\sum_{i}^{N}\left(y_{i}^{k}+p_{i}^{k}+\epsilon \right)},\;$$

where *N* represents the number of pixels, ϵ is a small positive number to avoid zero division, and the numerical range is set as $y_{i}^{k}\in \left\{0,1\right\}$ and $p_{i}^{k}\in \left[0,1\right]$. Here, the Dice loss is used for the prediction heads of lane and lane marking segmentation branches.

#### 3.5.2. Focal-Style Loss Weighting

To further alleviate the data imbalance problem in both lane marking and lane segmentation, with the inspiration of the focal loss [52], we propose a weighting process for both the lane and lane marking segmentation loss. The intuition of this loss weighting is to dynamically adjust weights for pixel-wise segmentation results so that hard pixels can be emphasized by assigning them larger back-propagated gradients during training. Reusing the semantic labels and predicted probabilities defined in Equation (1), the weight for the *i*-th pixel is defined as

(2) $$W_{i}=1+\frac{\alpha }{C}\sum_{k=1}^{C}\left(y_{i}^{k}{\left(1-p_{i}^{k}\right)}^{\gamma }+\left(1-y_{i}^{k}\right){\left(p_{i}^{k}\right)}^{\gamma }\right),\;$$

where α and γ are hyperparameters.

#### 3.5.3. Cross-IoU Loss

The Intersection over Union (IoU) [53] metric is commonly utilized to evaluate the pixel-level prediction performance in terms of tasks of object detection and segmentation, defined as

(3) $$IoU=\frac{TP}{FP+TP+FN},$$

where TP, FP, and FN denote the true positive, the false positive, and the false negative pixel counts, respectively. Considering the fact that the lane and lane marking are closely connected but do not overlap with each other, we also employ the IoU loss to suppress the overlapping between them and thus obtain the Cross-IoU Loss, dubbed as CIoU and computed by

(4) $$\begin{array}{cc} L_{CIoU}= & \frac{R_{l}\cap {\hat{R}}_{m}}{R_{l}\cup {\hat{R}}_{m}}\\ + & \frac{R_{m}\cap {\hat{R}}_{l}}{R_{m}\cup {\hat{R}}_{l}}, \end{array}$$

where $R_{l}$ and $R_{m}$ denote the pixel area of predicted lane and lane marking, respectively. The superscript ^ refers to the corresponding groundtruth.

#### 3.5.4. Total Learning Loss

To train the proposed multi-task architecture, the total learning loss is defined as

(5) $$\begin{array}{cc} L_{t}= & w_{m}\sum_{i}^{N}W_{i,m}L_{i,m}+w_{l}\sum_{i}^{N}W_{i,l}L_{i,l}\\ \; & +w_{CIoU}L_{CIoU}+w_{aux,l}L_{aux,l}+w_{aux,m}L_{aux,m}, \end{array}$$

where $L_{aux,l}$ and $L_{aux,m}$ denote the auxiliary losses (in the Cross Entropy form) for the lane and lane marking segmentation branches, respectively. $L_{i,l}$ and $L_{i,m}$ denote the pixel-wise Dice loss for the outputs of the lane and lane marking prediction heads, and $W_{i,l}$ and $W_{i,m}$ are the proposed Focal-style loss weightings. Other trade-off factors $w_{l}$, $w_{m}$, $w_{CIoU}$, $w_{aux,l}$ and $w_{aus,m}$ are hyperparameters.

## 4. Experiments and Evaluation

### 4.1. Implementation Details

#### 4.1.1. Datasets

To train and validate our proposed architecture, it requires annotations for both lane markings and drivable lane areas in the same dataset. Among the public benchmarks, the Berkeley DeepDrive (BDD100K) dataset [16] is the only one that can provide such kinds of annotations, which are also with a high variety in traffic scenes including illumination change and complex weathers. Thus, we choose it for our experiments as it supports the multi-task learning of our approach. The BDD100K consists of 100 K images in a size of 1280 × 720 pixels, in which 70 K images are used as a training set, 10 K images are used as a validation set, and 20 K images are used as a test set. Since the test labels are not publicly accessible and the evaluation of lane and lane marking detection is also unavailable on the server, we opt to evaluate the proposed method on the validation set.

#### 4.1.2. Metric

As mentioned above, we define both lane and lane marking detection as semantic segmentation tasks. Following the common protocol [38], we evaluate the accuracy of segmented drivable area and background using the mean IoU (mIoU) metric for lane detection while the lane marking segmentation is only evaluated by the IoU metric, to exclude the influence of background pixels, which occupy over 90% of the image. Specifically, given the predicted mask $M_{p,i}$ and the groundtruth mask $M_{g,i}$ of image *i*, the true positives count TP, true negative count TN, false negative count FN and false positive count FP are computed as

(6) $$TP=\sum_{i}\left|\right|M_{p,i}\cdot M_{g,i}{\left|\right|}_{0},$$

(7) $$TN=\sum_{i}\left|\right|\left(1-M_{p,i}\right)\cdot \left(1-M_{g,i}\right){\left|\right|}_{0},$$

(8) $$FN=\sum_{i}\left|\right|\left(1-M_{p,i}\right)\cdot M_{g,i}{\left|\right|}_{0},$$

(9) $$FP=\sum_{i}\left|\right|M_{p,i}\cdot \left(1-M_{g,i}\right){\left|\right|}_{0}.$$

The IoU metric can thus be calculated by referring to Equation (3). Additionally, we also evaluate the lane marking segmentation by the Pixel Accuracy (PA) metric, which can be calculated as

(10) $$PA=\frac{TP+TN}{TP+TN+FP+FN}.$$

#### 4.1.3. Training

For a fair comparison, the images of the BDD100K dataset are resized into a resolution of 640 × 384 pixels as the input of our architecture, which is the same size as used in other state-of-the-art approaches. In the experiment, the data augmentation tricks including the random rotation, random cutout, photometric distortions, and random horizontal flip are also adopted in the training. We use the Adam optimizer with a learning rate initialized to $2\times 10^{-4}$ and a weight decay set to $1\times 10^{-5}$. Other parameters follow the default settings. For the total loss, we set the weights $w_{l}$, $w_{m}$, $w_{iou}$, $w_{aux,l}$ and $w_{aux,m}$ to 1.0, 0.1, 0.1, 0.01 and 0.01, respectively. The hyperparameters of Focal-style loss weighting are empirically set to α=0.5 and γ=1. The model is trained with a batch-size of 8 in 150 epochs. All the experiments are carried out on a computer platform with a CPU of 2.5 GHz and a GPU of NVIDIA RTX 3090. Furthermore, for comparison with state-of-the-art methods, the ConvNeXt-Small [48] is chosen as our backbone.

**Figure 5:**
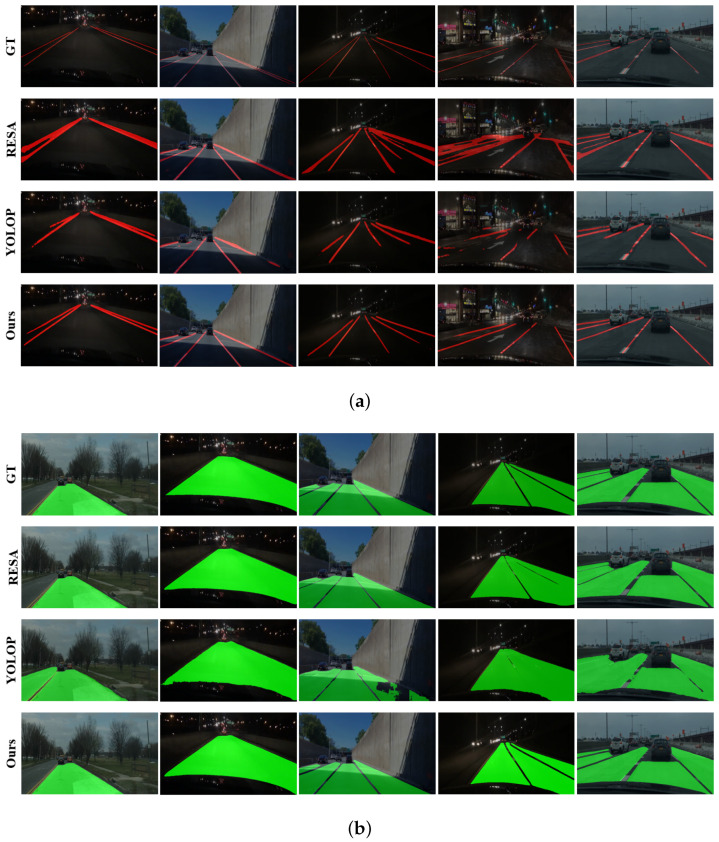
Visualized lane and lane marking detection by compared methods. “GT” is short for the groundtruth. (**a**) Lane marking detection results; (**b**) lane detection results.

Here, we evaluate our proposed architecture on the lane and lane marking detection by comparing it with other state-of-the-art approaches. For evaluation on the lane marking detection, we choose the ENet [54], SCNN [9], and ENet-SAD [6] as comparison sets. For comparison on the lane segmentation, we choose the ERFNet [55], MultiNet [41], DLT-Net [42], and PSPNet [34]. Additionally, we compare our architecture with the efficient feature aggregator RESA [10] and recently proposed multi-task approach YOLOP [49] in both evaluations (also visualized in Figure 5). Note that the RESA is originally designed for lane marking segmentation while we add an additional output head so that it is capable to segment the lane area.

### 4.2. Comparison with State-of-the-Arts

#### 4.2.1. Lane Marking Detection

The lane marking labels in the BDD100K dataset are annotated with sets of points, which is troublesome to directly use them. Thus, we follow the work [49] to substitute the two-line annotation with one center line and dilate the line width in the training to 8 pixels and keep it in the test as 2 pixels. The lane marking detection results are listed in Table 1.

As depicted in the table, our method outperforms ENet, SCNN, ENet-SAD, RESA, YOLOP by 47.49%, 45.82%, 45.05%, 20.25%, 11.11% in terms of accuracy and 17.89%, 16.69%, 16.51%, 15.82%, 6.33% in terms of IoU. The ENet adopts an early downsampling strategy for the encoding features to obtain a very fast processing of 100 frames per second (fps), yet leading to decreased detection accuracy. Based the shape characteristics of lane marking, the SCNN replaces the ordinary layer-by-layer convolutions with the slice-by-slice convolutions to pass information between rows and columns, resulting in a slow forward computation. The ENet-SAD introduces the paradigm of knowledge distillation on the basis of ENet, through which the intermediate encoding features are enhanced. However, limited to the architecture of ENet, the accuracy of ENet-SAD is not much improved. The RESA adopts an operation named Recurrent Feature-Shift to pass information between rows and columns, which is more accurate and efficient compared to slice-by-slice convolutions in SCNN. The YOLOP sets up multi-task prediction heads based on the pretraining of YOLOv5, which achieves better detection accuracy and real-time performance. However, it does not consider the geometric constraints between lane and lane markings and thus its prediction is still less optimal. Examples of the lane marking detection are visualized in Figure 5a. Note that we only visualize the results of those approaches whose codes are publicly available. As shown in Figure 5b, the RESA achieves a poor performance in the night driving scenario due to the limited visual information. Despite integrated with multi-tasks, there are still many discontinuities in the lane marking detection results of YOLOP, implying an insufficient learning about the interactive information between tasks. In comparison, our method predicts the lane markings more accurately.

#### 4.2.2. Lane Detection

For utility and briefness, the lane segmentation labels {direct, alternative} in the BDD100K dataset are merged into the label {lane}. Here, the lane area segmentation task is simplified to distinguish between the lane areas and the background areas in the image, resulting in two class labels. The lane detection results of compared approaches are listed in Table 2. From the reported results, we can observe that our method outperforms the ERFNet, MultiNet, DLT-Net, PSPNet, RESA and YOLOP by 23.02%, 20.12%, 19.62%, 2.12%, 2.42% and 0.22% in terms of mIoU. The ERFNet runs faster than the MultiNet, DLT-Net and PSPNet due to its residual connections and factorized convolutions for semantic segmentation. But it has only a simple encoder and a decoder, yielding an ordinary detection performance. The MultiNet handles the tasks of vehicle detection, scene classification, and lane detection at the same time and outperforms ERFNet by 2.9%. The DLT-Net adopts context tensors to share the information between subtask decoders, which results in an improved lane detection accuracy than MultiNet. The PSPNet consists of one encoder, one decoder, and one Pyramid Pooling module which incorporates multi-scale information and improves the lane detection performance. The RESA also achieves good results on the lane detection task by aggregating spatial information of intermediate features. Due to the advantages of the multi-task form and the pretrained backbone of YOLOv5, the YOLOP surpasses previous methods on the lane detection task. Several lane detection results are visualized in Figure 5a. Interestingly, although our method has a similar mIoU to YOLOP in the lane detection task, the visualization shows that our proposed method performs a more accurate and robust detection in several driving scenarios.

Moreover, the processing efficiency of our architecture is 26 fps, which is still appropriate for real-time applications.

### 4.3. Exploration on Interaction Learning Modules

Here, we explore the effectiveness of interaction-learning-related modules utilized in our architecture such as the DFF module, the Cross-Context module and the Focal-style loss weighting. For a qualitative impression, we also adopt the Grad-CAM [56] tool to visualize features extracted from the intermediate layer of those modules.

#### 4.3.1. DFF Module

**Figure 6:**
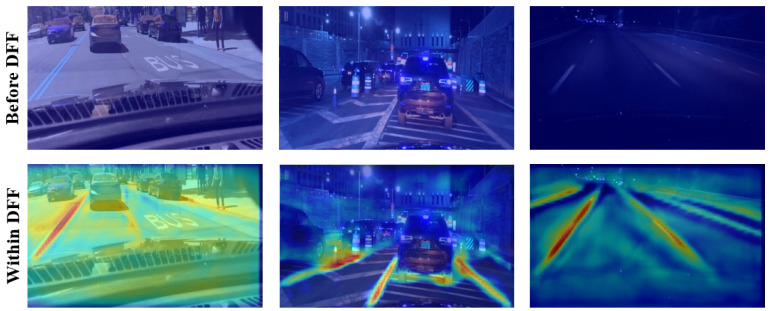
Activation map visualization for the DFF module at the layer before (**top**) and at the intermediate convolution layer within this module (**bottom**).

In an effort to verify the DFF module, we choose the network layer before the DFF and the intermediate convolution layer that generates the offsets for the deformable convolution to output the Grad-CAM activation map. The visualized activation maps “before” and “within” the DFF module are shown in Figure 6. Obviously, the model pays homogeneous attention to each pixel in the image before applying the DFF module, while with the DFF processing, the model focuses mainly on the features located on the lane markings. This fact verifies the capability of the DFF module in guiding the model learning discriminative features with the interaction information.

#### 4.3.2. Cross-Context Module

**Figure 7:**
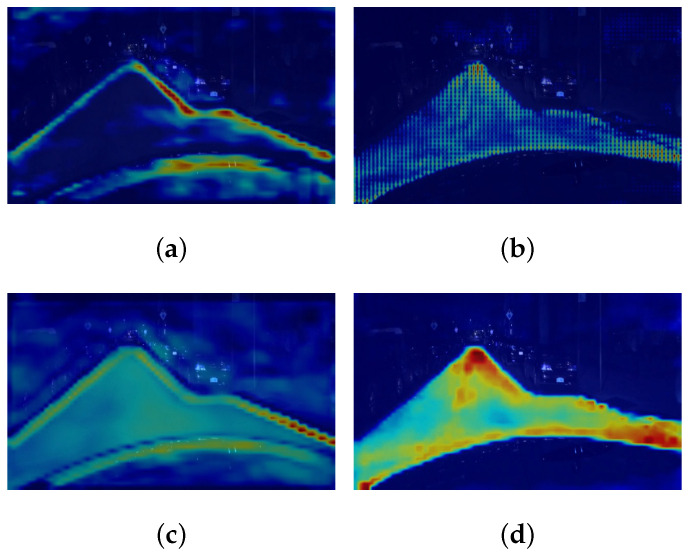
Activation map visualization for the Cross-Context module: (**a**) at the input layer of the lane marking prediction head; (**b**) at the input layer of the lane prediction head; (**c**) at the output layer of the Cross-Context module, which is added to the lane marking prediction head; (**d**) at the output layer of the Cross-Context module, which is added to the lane prediction head.

For the lane and lane marking prediction heads, we select the input layer of decoder and the output layer of Cross-Context module, respectively, to output the Grad-CAM activation map, as visualized in Figure 7a–d. It is worth noting that Figure 7c just depicts the feature map to be added to the input of lane marking prediction head while Figure 7d shows the feature map added to the other one. Thus, it is obviously that the Cross-Context Module can enhance the input features of both lane and lane marking prediction heads by reciprocal information transferring.

#### 4.3.3. Focal-Style Loss Weighting

**Figure 8:**
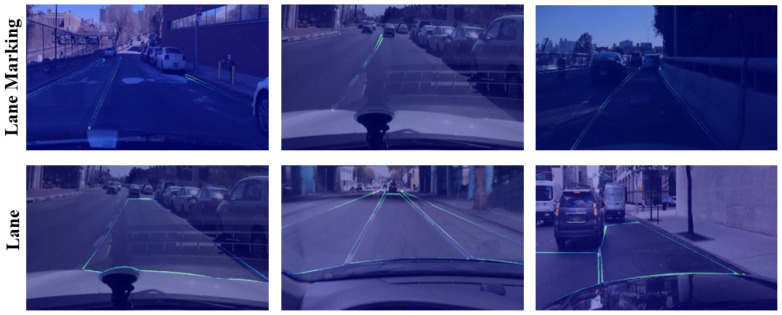
Visualization of the Focal-style loss weighting in heatmap for lane marking detection (**top**) and lane area segmentation (**bottom**). The brighter the color of heat map is, the greater the loss weight will be, and vice versa.

The weight map generated by the Focal-style loss weighting is shown in Figure 8. From the visualization, we can confirm that the weight map sets larger weights on the lane markings and lane boundaries. Under this circumstance, the model pays more attention to the corresponding pixels during training, which helps the inference of lane and lane markings and improves the detection accuracy, thus verifying the effectiveness of Focal-style loss weighting.

For the quantitative evaluation of all interaction learning modules, we provide an ablation study. For the baseline, we design a model with a single-branch backbone (i.e., 1× scale of input image), a single neck, and two separate task heads. For training efficiency, we adopt the ResNet-18 as backbone. Subsequently, we gradually integrate the encoder with multi-scale inputs, the DFF Module, the Cross-Context module in the decoder, the Cross-IoU loss, and the Focal-style loss weighting into the baseline. We report the performance of above integrated versions in Table 3. As can be seen, the detection accuracy of both lane and lane marking gradually increases with more modules integrated. The performance gain brought by the Cross-Context module, the CIoU loss and the Focal-style loss weighting are relative large, which is over 4% on the lane marking IoU metric and over 2% on the lane mIoU metric, further demonstrating the great advantages by interaction learning.

### 4.4. Further Exploration on Architecture Design

In an effort to further explore the impact of different backbone paradigms on the detection performance of our architecture, we select a group of backbone networks for comparison. Concretely, we set up the ResNet18, ResNet34, ConvNeXt-tiny, and ConvNeXt-small as the backbone and other modules remain unchanged. It is worth noting that the 1/32 downsampling layers in the naive ResNet and ConvNeXt discard too much information, which degrades the performance of following modules. Thus, for the ResNet, we replace the ordinary convolution in the downsampling layer of the C4 and C5 stage with the atrous convolution, which can expand the receptive field of the network while maintaining a higher spatial resolution. The ConvNeXt can thus be configured referring to Section 3.2.2. The results are listed in Table 4. As to be seen, since the ConvNeXt has a well-designed architecture with more network parameters, its accuracy on both lane and lane marking detection increases compared to the ResNet while the ResNet shows a lower computation amount and a faster real-time performance due to its fewer parameters.

## 5. Conclusions

In this paper, we put forward a novel multi-task framework for vision-based lane and lane marking detection on the road by introducing the interaction learning of their tight spatial correlation, which is persistently neglected in existing researches. The efficient learning of the interaction between the lane and lane marking information is achieved by three novel modules, i.e., the Deformable Feature Fusion Module for feature encoding, the Cross-Context Module for information decoding, the Cross-IoU loss and the Focal-style loss weighting for robust training. The effectiveness of each module has been validated based on throughout analysis of comprehensive experiments on the challenging BDD100K dataset. Therefore, the neglected spatial correlation between lane and lane marking in previous works has been proven essential to the improvement of detection robustness. Our proposed architecture also surpasses state-of-the-art approaches on both lane and lane detection tasks at a processing speed of 26 fps, which is promising for applications with real-time requirements. In the future work, we will introduce the interactive attention learning into the transformer architecture and further reduce the computation cost of attention estimation. We will also extend our method to various weather conditions and validate it with more new datasets, since the number of public datasets currently supporting both lane and lane marking detection tasks is still limited. Moreover, we will investigate the joint learning of both dynamic elements (e.g., vehicles, pedestrians) and static elements (e.g., lanes, lane markings) to improve the detection accuracy.

## Figures and Tables

**Table 1:** Comparison results on lane marking detection.

Network	Accuracy (%) ↑	IoU (%) ↑	Speed (fps) ↑
ENet [54]	34.12	14.64	**100**
SCNN [9]	35.79	15.84	19.8
ENet-SAD [6]	36.56	16.02	50.6
RESA [10]	61.26	16.71	47.4
YOLOP [49]	70.50	26.20	41
Ours	**81.61**	**32.53**	26

**Table 2:** Comparison results on lane detection.

Network	mIoU (%) ↑	Speed (fps) ↑
ERFNet [55]	68.7	22.8
MultiNet [41]	71.6	8.6
DLT-Net [42]	72.1	9.3
PSPNet [34]	89.6	11.1
RESA [10]	89.3	**47.4**
YOLOP [49]	91.5	41
Ours	**91.72**	26

**Table 3:** Performance exploration of proposed modules on the BDD100K dataset. The baseline is the model with a single-scale input encoder and two separate task decoders.

Baseline	Multi-Scale	DFF	Cross-Context	CIoU	Focal-Style	Lane Marking IoU (%) ↑	Lane mIoU (%) ↑
✓						19.14	87.40
✓	✓					21.49 (+2.35)	87.47 (+0.07)
✓	✓	✓				21.83 (+2.69)	88.85 (+1.45)
✓	✓	✓	✓			23.2 (+4.06)	89.47 (+2.07)
✓	✓	✓	✓	✓		23.49 (+4.35)	89.54 (+2.14)
✓	✓	✓	✓	✓	✓	**23.96 (+4.82)**	**90.07 (+2.67)**

**Table 4:** Comparison of model performance with different backbone paradigms on the BDD100K dataset.

Backbone	Lane Marking IoU (%) ↑	Lane mIoU (%) ↑	Params (M) ↓	FLOPs (G) ↓	Speed (fps) ↑
ResNet-18	30.39	90.54	**17.05**	**89.83**	**58**
ResNet-34	30.46	90.61	27.16	139.46	40
ConvNeXt-tiny	31.48	91.29	18.35	96.52	39
ConvNeXt-small	**32.53**	**91.72**	39.97	200.07	26

## Data Availability

The datasets generated and analysed during the current study are available in the [BDD100K] repository. [https://bdd-data.berkeley.edu/, accessed on 1 December 2022].
